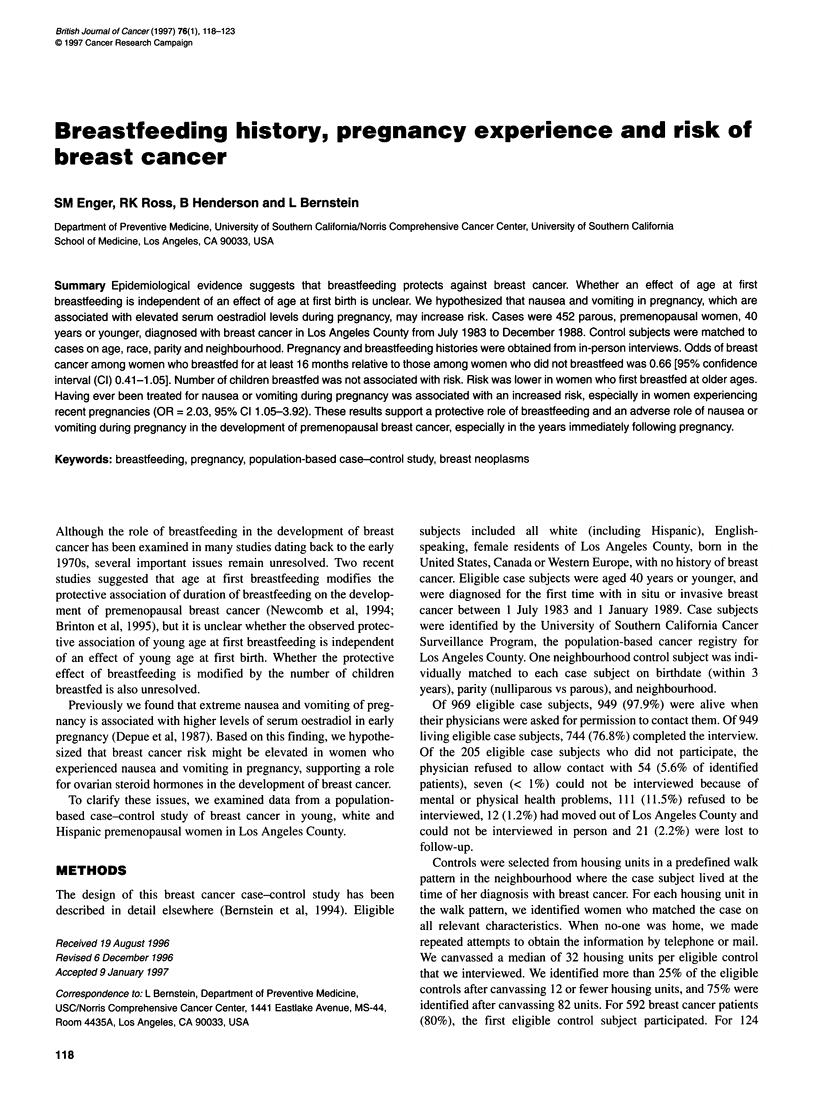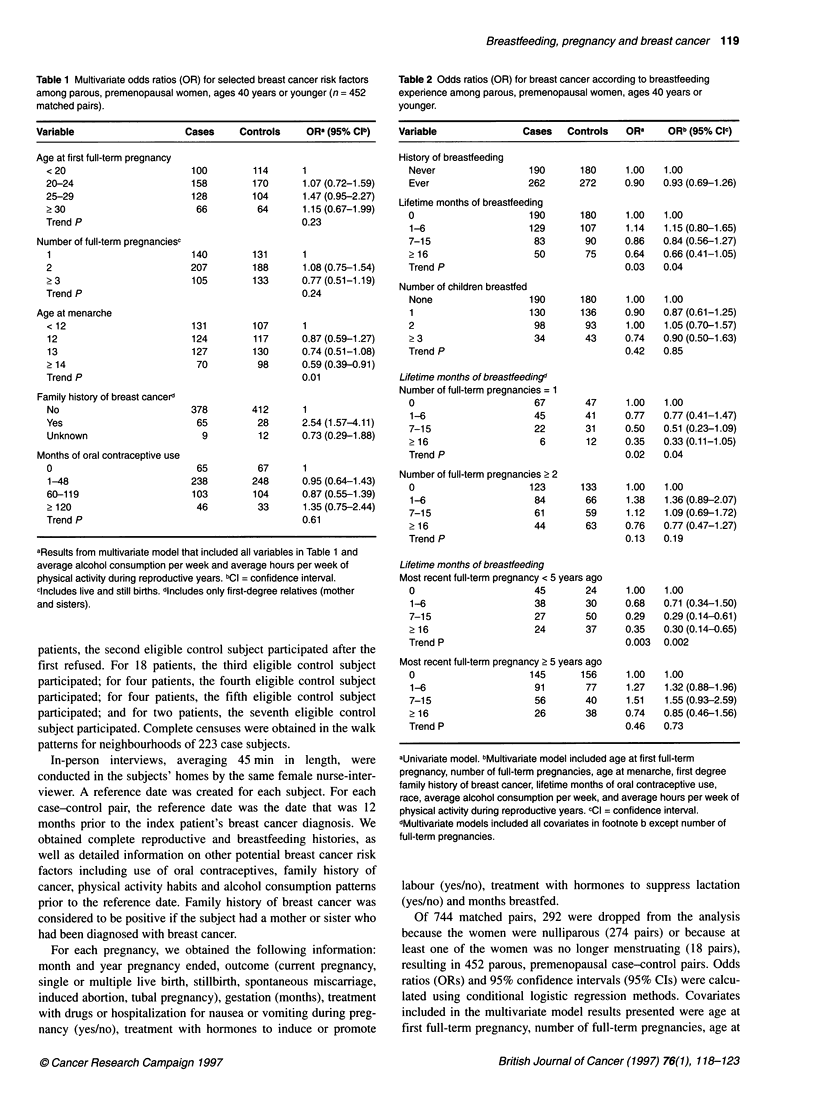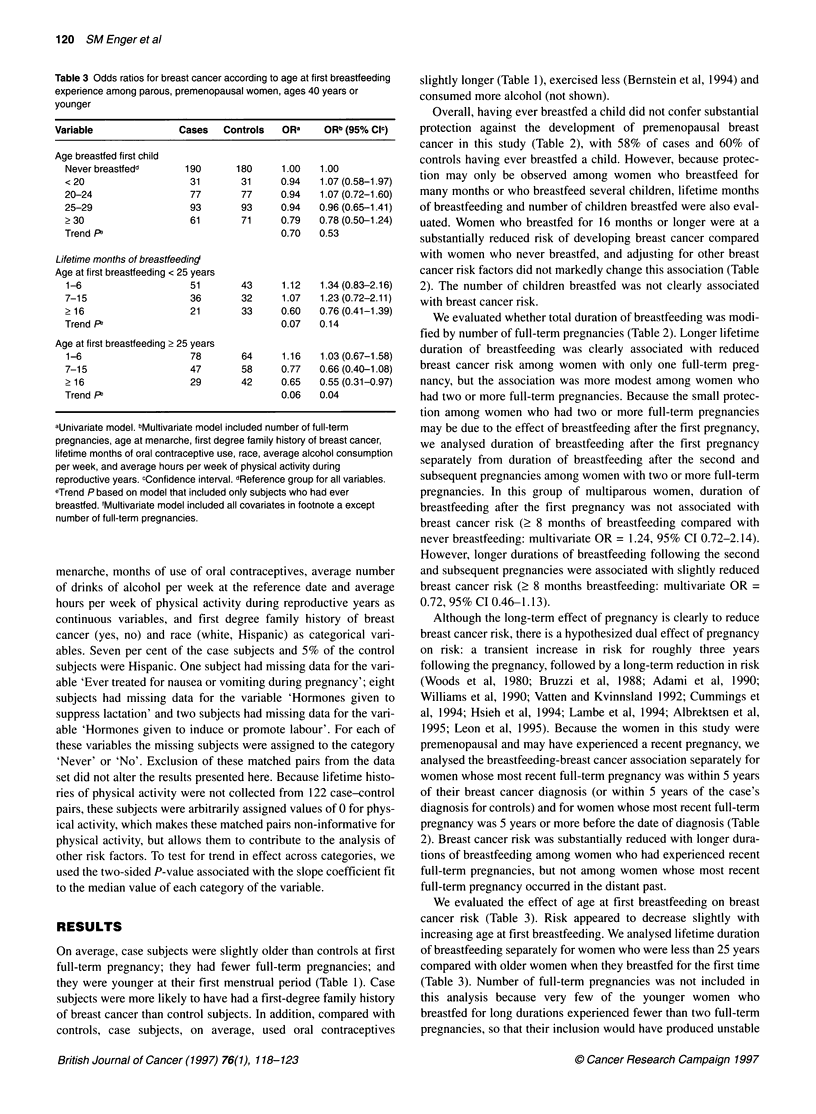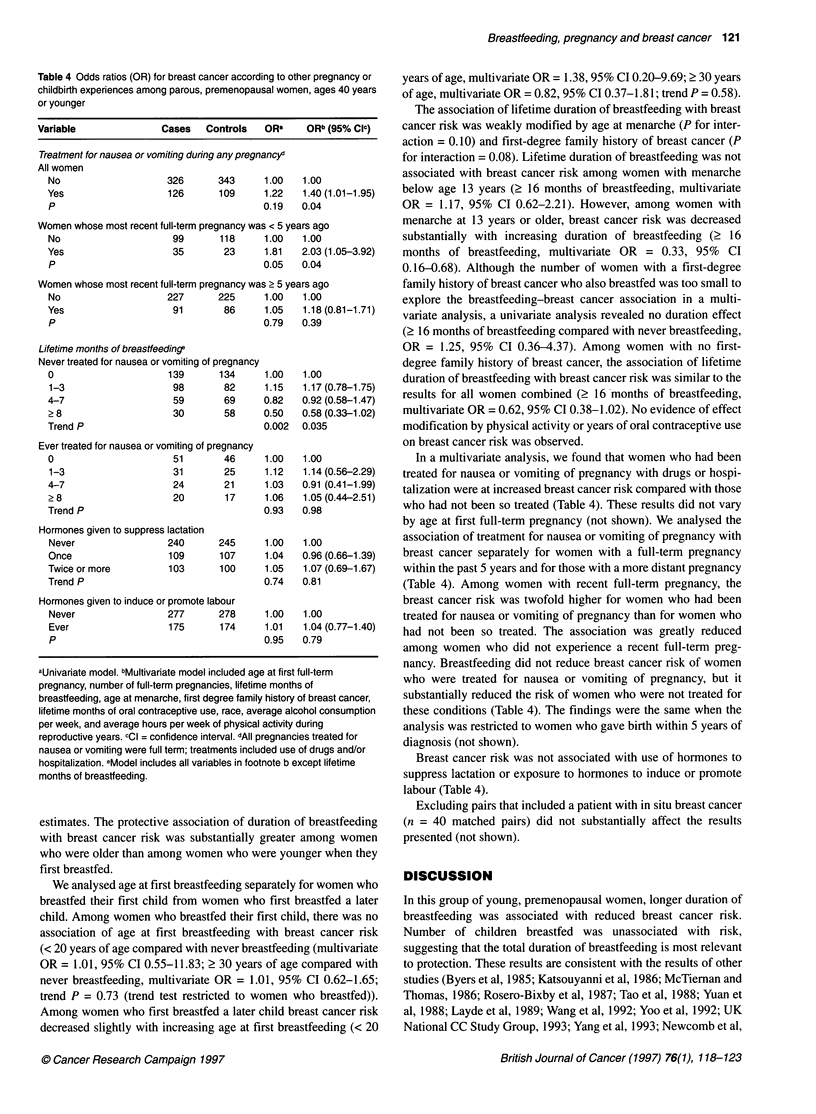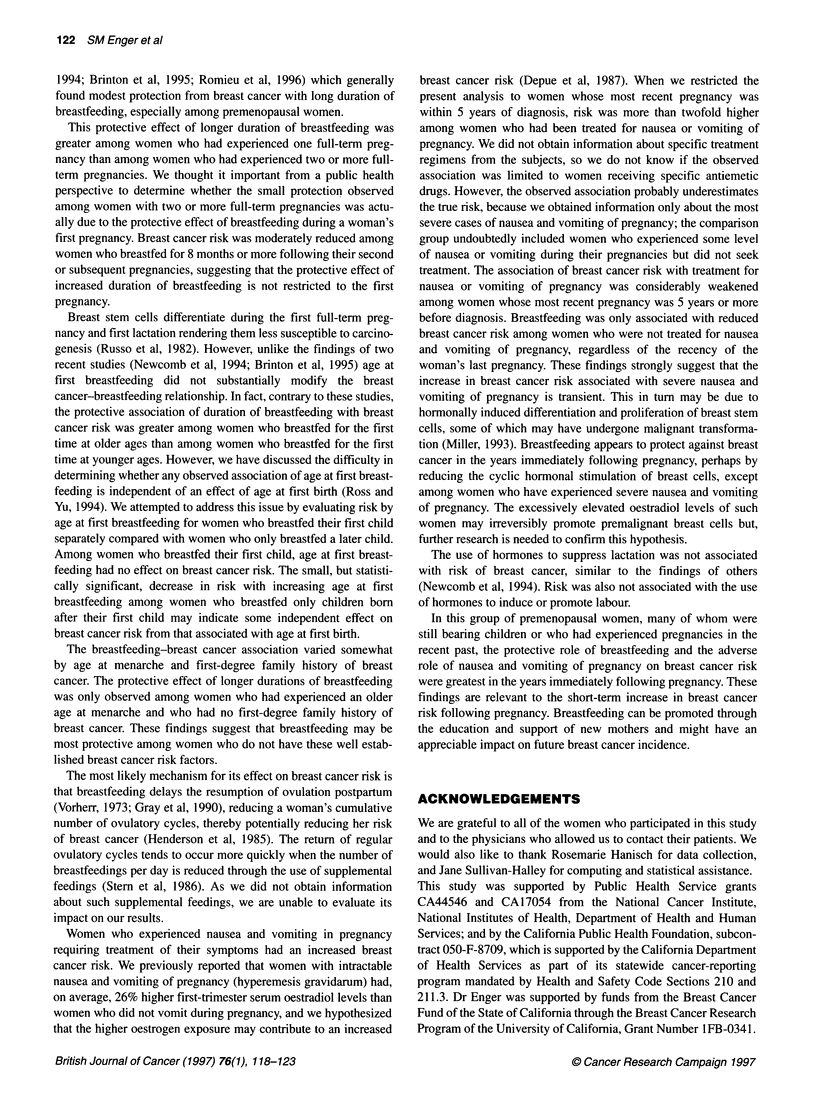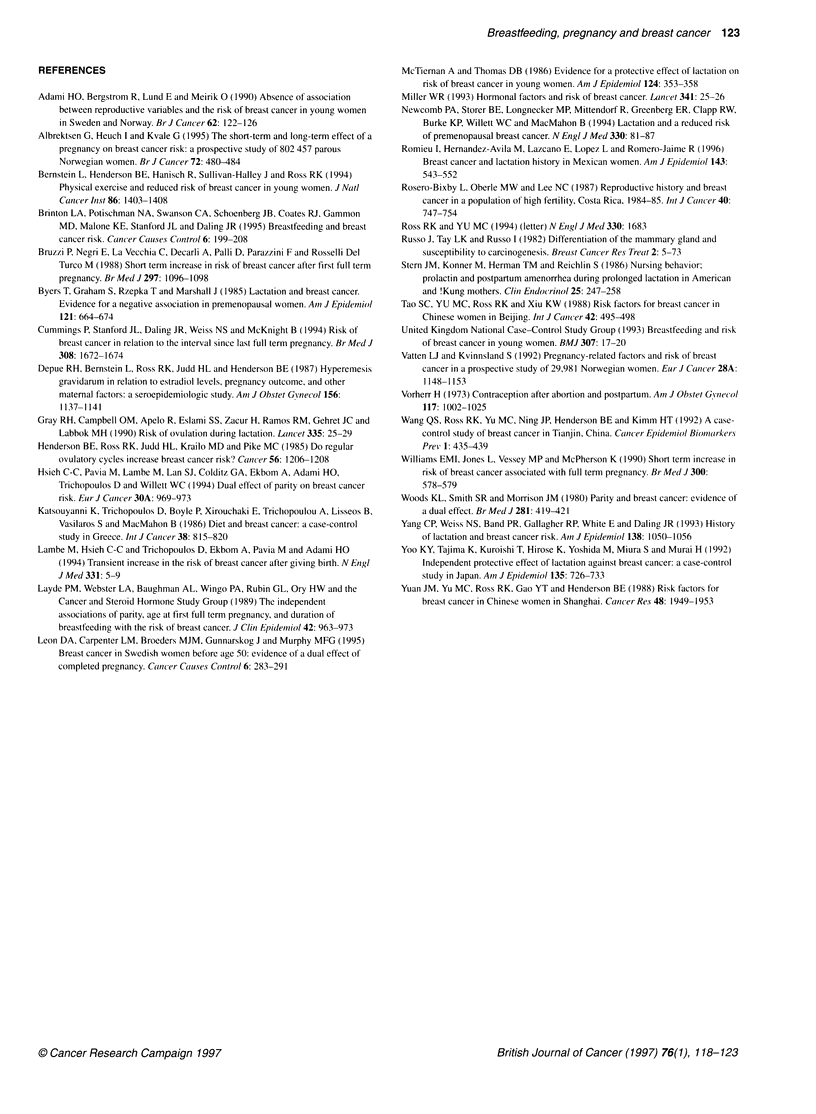# Breastfeeding history, pregnancy experience and risk of breast cancer.

**DOI:** 10.1038/bjc.1997.346

**Published:** 1997

**Authors:** S. M. Enger, R. K. Ross, B. Henderson, L. Bernstein

**Affiliations:** Department of Preventive Medicine, University of Southern California/Norris Comprehensive Cancer Center, School of Medicine, Los Angeles 90033, USA.

## Abstract

Epidemiological evidence suggests that breastfeeding protects against breast cancer. Whether an effect of age at first breastfeeding is independent of an effect of age at first birth is unclear. We hypothesized that nausea and vomiting in pregnancy, which are associated with elevated serum oestradiol levels during pregnancy, may increase risk. Cases were 452 parous, premenopausal women, 40 years or younger, diagnosed with breast cancer in Los Angeles County from July 1983 to December 1988. Control subjects were matched to cases on age, race, parity and neighbourhood. Pregnancy and breastfeeding histories were obtained from in-person interviews. Odds of breast cancer among women who breastfed for at least 16 months relative to those among women who did not breastfeed was 0.66 [95% confidence interval (CI) 0.41-1.05]. Number of children breastfed was not associated with risk. Risk was lower in women who first breastfed at older ages. Having ever been treated for nausea or vomiting during pregnancy was associated with an increased risk, especially in women experiencing recent pregnancies (OR = 2.03, 95% CI 1.05-3.92). These results support a protective role of breastfeeding and an adverse role of nausea or vomiting during pregnancy in the development of premenopausal breast cancer, especially in the years immediately following pregnancy.


British Journal of Cancer (1997) 76(1), 118–123
© 1997 Cancer Research Campaign

### Breastfeeding history, pregnancy experience and risk of breast cancer

**SM Enger, RK Ross, B Henderson and L Bernstein**

Department of Preventive Medicine, University of Southern California/Norris Comprehensive Cancer Center, University of Southern California School of Medicine, Los Angeles, CA 90033, USA

**Summary** Epidemiological evidence suggests that breastfeeding protects against breast cancer. Whether an effect of age at first breastfeeding is independent of an effect of age at first birth is unclear. We hypothesized that nausea and vomiting in pregnancy, which are associated with elevated serum oestradiol levels during pregnancy, may increase risk. Cases were 452 parous, premenopausal women, 40 years or younger, diagnosed with breast cancer in Los Angeles County from July 1983 to December 1988. Control subjects were matched to cases on age, race, parity and neighbourhood. Pregnancy and breastfeeding histories were obtained from in-person interviews. Odds of breast cancer among women who breastfed for at least 16 months relative to those among women who did not breastfeed was 0.66 [95% confidence interval (CI) 0.41–1.05]. Number of children breastfed was not associated with risk. Risk was lower in women who first breastfed at older ages. Having ever been treated for nausea or vomiting during pregnancy was associated with an increased risk, especially in women experiencing recent pregnancies (OR = 2.03, 95% CI 1.05–3.92). These results support a protective role of breastfeeding and an adverse role of nausea or vomiting during pregnancy in the development of premenopausal breast cancer, especially in the years immediately following pregnancy.

**Keywords:** breastfeeding, pregnancy, population-based case–control study, breast neoplasms

Although the role of breastfeeding in the development of breast cancer has been examined in many studies dating back to the early 1970s, several important issues remain unresolved. Two recent studies suggested that age at first breastfeeding modifies the protective association of duration of breastfeeding on the development of premenopausal breast cancer (Newcomb et al, 1994; Brinton et al, 1995), but it is unclear whether the observed protective association of young age at first breastfeeding is independent of an effect of young age at first birth. Whether the protective effect of breastfeeding is modified by the number of children breastfed is also unresolved.

Previously we found that extreme nausea and vomiting of pregnancy is associated with higher levels of serum oestradiol in early pregnancy (Depue et al, 1987). Based on this finding, we hypothesized that breast cancer risk might be elevated in women who experienced nausea and vomiting in pregnancy, supporting a role for ovarian steroid hormones in the development of breast cancer.

To clarify these issues, we examined data from a population-based case–control study of breast cancer in young, white and Hispanic premenopausal women in Los Angeles County.

### METHODS

The design of this breast cancer case–control study has been described in detail elsewhere (Bernstein et al, 1994). Eligible subjects included all white (including Hispanic), English-speaking, female residents of Los Angeles County, born in the United States, Canada or Western Europe, with no history of breast cancer. Eligible case subjects were aged 40 years or younger, and were diagnosed for the first time with in situ or invasive breast cancer between 1 July 1983 and 1 January 1989. Case subjects were identified by the University of Southern California Cancer Surveillance Program, the population-based cancer registry for Los Angeles County. One neighbourhood control subject was individually matched to each case subject on birthdate (within 3 years), parity (nulliparous vs parous), and neighbourhood.

Of 969 eligible case subjects, 949 (97.9%) were alive when their physicians were asked for permission to contact them. Of 949 living eligible case subjects, 744 (76.8%) completed the interview. Of the 205 eligible case subjects who did not participate, the physician refused to allow contact with 54 (5.6% of identified patients), seven (< 1%) could not be interviewed because of mental or physical health problems, 111 (11.5%) refused to be interviewed, 12 (1.2%) had moved out of Los Angeles County and could not be interviewed in person and 21 (2.2%) were lost to follow-up.

Controls were selected from housing units in a predefined walk pattern in the neighbourhood where the case subject lived at the time of her diagnosis with breast cancer. For each housing unit in the walk pattern, we identified women who matched the case on all relevant characteristics. When no-one was home, we made repeated attempts to obtain the information by telephone or mail. We canvassed a median of 32 housing units per eligible control that we interviewed. We identified more than 25% of the eligible controls after canvassing 12 or fewer housing units, and 75% were identified after canvassing 82 units. For 592 breast cancer patients (80%), the first eligible control subject participated. For 124

*Received 19 August 1996*
*Revised 6 December 1996*
*Accepted 9 January 1997*

*Correspondence to:* L Bernstein, Department of Preventive Medicine, USC/Norris Comprehensive Cancer Center, 1441 Eastlake Avenue, MS-44, Room 4435A, Los Angeles, CA 90033, USA

**Table 1** Multivariate odds ratios (OR) for selected breast cancer risk factors among parous, premenopausal women, ages 40 years or younger ($n$ = 452 matched pairs).

| Variable | Cases | Controls | OR[a] (95% CI[b]) |
|---|---|---|---|
| Age at first full-term pregnancy | | | |
| < 20 | 100 | 114 | 1 |
| 20–24 | 158 | 170 | 1.07 (0.72–1.59) |
| 25–29 | 128 | 104 | 1.47 (0.95–2.27) |
| ≥ 30 | 66 | 64 | 1.15 (0.67–1.99) |
| Trend *P* | | | 0.23 |
| Number of full-term pregnancies[c] | | | |
| 1 | 140 | 131 | 1 |
| 2 | 207 | 188 | 1.08 (0.75–1.54) |
| ≥ 3 | 105 | 133 | 0.77 (0.51–1.19) |
| Trend *P* | | | 0.24 |
| Age at menarche | | | |
| < 12 | 131 | 107 | 1 |
| 12 | 124 | 117 | 0.87 (0.59–1.27) |
| 13 | 127 | 130 | 0.74 (0.51–1.08) |
| ≥ 14 | 70 | 98 | 0.59 (0.39–0.91) |
| Trend *P* | | | 0.01 |
| Family history of breast cancer[d] | | | |
| No | 378 | 412 | 1 |
| Yes | 65 | 28 | 2.54 (1.57–4.11) |
| Unknown | 9 | 12 | 0.73 (0.29–1.88) |
| Months of oral contraceptive use | | | |
| 0 | 65 | 67 | 1 |
| 1–48 | 238 | 248 | 0.95 (0.64–1.43) |
| 60–119 | 103 | 104 | 0.87 (0.55–1.39) |
| ≥ 120 | 46 | 33 | 1.35 (0.75–2.44) |
| Trend *P* | | | 0.61 |

[a]Results from multivariate model that included all variables in Table 1 and average alcohol consumption per week and average hours per week of physical activity during reproductive years. [b]CI = confidence interval. [c]Includes live and still births. [d]Includes only first-degree relatives (mother and sisters).

patients, the second eligible control subject participated after the first refused. For 18 patients, the third eligible control subject participated; for four patients, the fourth eligible control subject participated; for four patients, the fifth eligible control subject participated; and for two patients, the seventh eligible control subject participated. Complete censuses were obtained in the walk patterns for neighbourhoods of 223 case subjects.

In-person interviews, averaging 45 min in length, were conducted in the subjects' homes by the same female nurse-interviewer. A reference date was created for each subject. For each case–control pair, the reference date was the date that was 12 months prior to the index patient's breast cancer diagnosis. We obtained complete reproductive and breastfeeding histories, as well as detailed information on other potential breast cancer risk factors including use of oral contraceptives, family history of cancer, physical activity habits and alcohol consumption patterns prior to the reference date. Family history of breast cancer was considered to be positive if the subject had a mother or sister who had been diagnosed with breast cancer.

For each pregnancy, we obtained the following information: month and year pregnancy ended, outcome (current pregnancy, single or multiple live birth, stillbirth, spontaneous miscarriage, induced abortion, tubal pregnancy), gestation (months), treatment with drugs or hospitalization for nausea or vomiting during pregnancy (yes/no), treatment with hormones to induce or promote labour (yes/no), treatment with hormones to suppress lactation (yes/no) and months breastfed.

Of 744 matched pairs, 292 were dropped from the analysis because the women were nulliparous (274 pairs) or because at least one of the women was no longer menstruating (18 pairs), resulting in 452 parous, premenopausal case–control pairs. Odds ratios (ORs) and 95% confidence intervals (95% CIs) were calculated using conditional logistic regression methods. Covariates included in the multivariate model results presented were age at first full-term pregnancy, number of full-term pregnancies, age at

**Table 2** Odds ratios (OR) for breast cancer according to breastfeeding experience among parous, premenopausal women, ages 40 years or younger.

| Variable | Cases | Controls | OR[a] | OR[b] (95% CI[c]) |
|---|---|---|---|---|
| History of breastfeeding | | | | |
| Never | 190 | 180 | 1.00 | 1.00 |
| Ever | 262 | 272 | 0.90 | 0.93 (0.69–1.26) |
| Lifetime months of breastfeeding | | | | |
| 0 | 190 | 180 | 1.00 | 1.00 |
| 1–6 | 129 | 107 | 1.14 | 1.15 (0.80–1.65) |
| 7–15 | 83 | 90 | 0.86 | 0.84 (0.56–1.27) |
| ≥ 16 | 50 | 75 | 0.64 | 0.66 (0.41–1.05) |
| Trend *P* | | | 0.03 | 0.04 |
| Number of children breastfed | | | | |
| None | 190 | 180 | 1.00 | 1.00 |
| 1 | 130 | 136 | 0.90 | 0.87 (0.61–1.25) |
| 2 | 98 | 93 | 1.00 | 1.05 (0.70–1.57) |
| ≥ 3 | 34 | 43 | 0.74 | 0.90 (0.50–1.63) |
| Trend *P* | | | 0.42 | 0.85 |
| *Lifetime months of breastfeeding*[d] | | | | |
| Number of full-term pregnancies = 1 | | | | |
| 0 | 67 | 47 | 1.00 | 1.00 |
| 1–6 | 45 | 41 | 0.77 | 0.77 (0.41–1.47) |
| 7–15 | 22 | 31 | 0.50 | 0.51 (0.23–1.09) |
| ≥ 16 | 6 | 12 | 0.35 | 0.33 (0.11–1.05) |
| Trend *P* | | | 0.02 | 0.04 |
| Number of full-term pregnancies ≥ 2 | | | | |
| 0 | 123 | 133 | 1.00 | 1.00 |
| 1–6 | 84 | 66 | 1.38 | 1.36 (0.89–2.07) |
| 7–15 | 61 | 59 | 1.12 | 1.09 (0.69–1.72) |
| ≥ 16 | 44 | 63 | 0.76 | 0.77 (0.47–1.27) |
| Trend *P* | | | 0.13 | 0.19 |
| *Lifetime months of breastfeeding* | | | | |
| Most recent full-term pregnancy < 5 years ago | | | | |
| 0 | 45 | 24 | 1.00 | 1.00 |
| 1–6 | 38 | 30 | 0.68 | 0.71 (0.34–1.50) |
| 7–15 | 27 | 50 | 0.29 | 0.29 (0.14–0.61) |
| ≥ 16 | 24 | 37 | 0.35 | 0.30 (0.14–0.65) |
| Trend P | | | 0.003 | 0.002 |
| Most recent full-term pregnancy ≥ 5 years ago | | | | |
| 0 | 145 | 156 | 1.00 | 1.00 |
| 1–6 | 91 | 77 | 1.27 | 1.32 (0.88–1.96) |
| 7–15 | 56 | 40 | 1.51 | 1.55 (0.93–2.59) |
| ≥ 16 | 26 | 38 | 0.74 | 0.85 (0.46–1.56) |
| Trend P | | | 0.46 | 0.73 |

[a]Univariate model. [b]Multivariate model included age at first full-term pregnancy, number of full-term pregnancies, age at menarche, first degree family history of breast cancer, lifetime months of oral contraceptive use, race, average alcohol consumption per week, and average hours per week of physical activity during reproductive years. [c]CI = confidence interval. [d]Multivariate models included all covariates in footnote b except number of full-term pregnancies.

© Cancer Research Campaign 1997

Table 3 Odds ratios for breast cancer according to age at first breastfeeding experience among parous, premenopausal women, ages 40 years or younger

| Variable | Cases | Controls | OR[a] | OR[b] (95% CI[c]) |
|---|---|---|---|---|
| Age breastfed first child | | | | |
| Never breastfed[d] | 190 | 180 | 1.00 | 1.00 |
| < 20 | 31 | 31 | 0.94 | 1.07 (0.58–1.97) |
| 20–24 | 77 | 77 | 0.94 | 1.07 (0.72–1.60) |
| 25–29 | 93 | 93 | 0.94 | 0.96 (0.65–1.41) |
| ≥ 30 | 61 | 71 | 0.79 | 0.78 (0.50–1.24) |
| Trend P[e] | | | 0.70 | 0.53 |
| *Lifetime months of breastfeeding*[f] | | | | |
| Age at first breastfeeding < 25 years | | | | |
| 1–6 | 51 | 43 | 1.12 | 1.34 (0.83–2.16) |
| 7–15 | 36 | 32 | 1.07 | 1.23 (0.72–2.11) |
| ≥ 16 | 21 | 33 | 0.60 | 0.76 (0.41–1.39) |
| Trend P[e] | | | 0.07 | 0.14 |
| Age at first breastfeeding ≥ 25 years | | | | |
| 1–6 | 78 | 64 | 1.16 | 1.03 (0.67–1.58) |
| 7–15 | 47 | 58 | 0.77 | 0.66 (0.40–1.08) |
| ≥ 16 | 29 | 42 | 0.65 | 0.55 (0.31–0.97) |
| Trend P[e] | | | 0.06 | 0.04 |

[a]Univariate model. [b]Multivariate model included number of full-term pregnancies, age at menarche, first degree family history of breast cancer, lifetime months of oral contraceptive use, race, average alcohol consumption per week, and average hours per week of physical activity during reproductive years. [c]Confidence interval. [d]Reference group for all variables. [e]Trend P based on model that included only subjects who had ever breastfed. [f]Multivariate model included all covariates in footnote a except number of full-term pregnancies.

menarche, months of use of oral contraceptives, average number of drinks of alcohol per week at the reference date and average hours per week of physical activity during reproductive years as continuous variables, and first degree family history of breast cancer (yes, no) and race (white, Hispanic) as categorical variables. Seven per cent of the case subjects and 5% of the control subjects were Hispanic. One subject had missing data for the variable 'Ever treated for nausea or vomiting during pregnancy'; eight subjects had missing data for the variable 'Hormones given to suppress lactation' and two subjects had missing data for the variable 'Hormones given to induce or promote labour'. For each of these variables the missing subjects were assigned to the category 'Never' or 'No'. Exclusion of these matched pairs from the data set did not alter the results presented here. Because lifetime histories of physical activity were not collected from 122 case–control pairs, these subjects were arbitrarily assigned values of 0 for physical activity, which makes these matched pairs non-informative for physical activity, but allows them to contribute to the analysis of other risk factors. To test for trend in effect across categories, we used the two-sided $P$-value associated with the slope coefficient fit to the median value of each category of the variable.

### RESULTS

On average, case subjects were slightly older than controls at first full-term pregnancy; they had fewer full-term pregnancies; and they were younger at their first menstrual period (Table 1). Case subjects were more likely to have had a first-degree family history of breast cancer than control subjects. In addition, compared with controls, case subjects, on average, used oral contraceptives slightly longer (Table 1), exercised less (Bernstein et al, 1994) and consumed more alcohol (not shown).

Overall, having ever breastfed a child did not confer substantial protection against the development of premenopausal breast cancer in this study (Table 2), with 58% of cases and 60% of controls having ever breastfed a child. However, because protection may only be observed among women who breastfeed for many months or who breastfeed several children, lifetime months of breastfeeding and number of children breastfed were also evaluated. Women who breastfed for 16 months or longer were at a substantially reduced risk of developing breast cancer compared with women who never breastfed, and adjusting for other breast cancer risk factors did not markedly change this association (Table 2). The number of children breastfed was not clearly associated with breast cancer risk.

We evaluated whether total duration of breastfeeding was modified by number of full-term pregnancies (Table 2). Longer lifetime duration of breastfeeding was clearly associated with reduced breast cancer risk among women with only one full-term pregnancy, but the association was more modest among women who had two or more full-term pregnancies. Because the small protection among women who had two or more full-term pregnancies may be due to the effect of breastfeeding after the first pregnancy, we analysed duration of breastfeeding after the first pregnancy separately from duration of breastfeeding after the second and subsequent pregnancies among women with two or more full-term pregnancies. In this group of multiparous women, duration of breastfeeding after the first pregnancy was not associated with breast cancer risk (≥ 8 months of breastfeeding compared with never breastfeeding: multivariate OR = 1.24, 95% CI 0.72–2.14). However, longer durations of breastfeeding following the second and subsequent pregnancies were associated with slightly reduced breast cancer risk (≥ 8 months breastfeeding: multivariate OR = 0.72, 95% CI 0.46–1.13).

Although the long-term effect of pregnancy is clearly to reduce breast cancer risk, there is a hypothesized dual effect of pregnancy on risk: a transient increase in risk for roughly three years following the pregnancy, followed by a long-term reduction in risk (Woods et al, 1980; Bruzzi et al, 1988; Adami et al, 1990; Williams et al, 1990; Vatten and Kvinnsland 1992; Cummings et al, 1994; Hsieh et al, 1994; Lambe et al, 1994; Albrektsen et al, 1995; Leon et al, 1995). Because the women in this study were premenopausal and may have experienced a recent pregnancy, we analysed the breastfeeding-breast cancer association separately for women whose most recent full-term pregnancy was within 5 years of their breast cancer diagnosis (or within 5 years of the case's diagnosis for controls) and for women whose most recent full-term pregnancy was 5 years or more before the date of diagnosis (Table 2). Breast cancer risk was substantially reduced with longer durations of breastfeeding among women who had experienced recent full-term pregnancies, but not among women whose most recent full-term pregnancy occurred in the distant past.

We evaluated the effect of age at first breastfeeding on breast cancer risk (Table 3). Risk appeared to decrease slightly with increasing age at first breastfeeding. We analysed lifetime duration of breastfeeding separately for women who were less than 25 years compared with older women when they breastfed for the first time (Table 3). Number of full-term pregnancies was not included in this analysis because very few of the younger women who breastfed for long durations experienced fewer than two full-term pregnancies, so that their inclusion would have produced unstable

 © Cancer Research Campaign 1997

**Table 4** Odds ratios (OR) for breast cancer according to other pregnancy or childbirth experiences among parous, premenopausal women, ages 40 years or younger

| Variable | Cases | Controls | OR[a] | OR[b] (95% CI[c]) |
|---|---|---|---|---|
| *Treatment for nausea or vomiting during any pregnancy*[d] | | | | |
| All women | | | | |
| No | 326 | 343 | 1.00 | 1.00 |
| Yes | 126 | 109 | 1.22 | 1.40 (1.01–1.95) |
| *P* | | | 0.19 | 0.04 |
| Women whose most recent full-term pregnancy was < 5 years ago | | | | |
| No | 99 | 118 | 1.00 | 1.00 |
| Yes | 35 | 23 | 1.81 | 2.03 (1.05–3.92) |
| *P* | | | 0.05 | 0.04 |
| Women whose most recent full-term pregnancy was ≥ 5 years ago | | | | |
| No | 227 | 225 | 1.00 | 1.00 |
| Yes | 91 | 86 | 1.05 | 1.18 (0.81–1.71) |
| *P* | | | 0.79 | 0.39 |
| *Lifetime months of breastfeeding*[e] | | | | |
| Never treated for nausea or vomiting of pregnancy | | | | |
| 0 | 139 | 134 | 1.00 | 1.00 |
| 1–3 | 98 | 82 | 1.15 | 1.17 (0.78–1.75) |
| 4–7 | 59 | 69 | 0.82 | 0.92 (0.58–1.47) |
| ≥ 8 | 30 | 58 | 0.50 | 0.58 (0.33–1.02) |
| Trend *P* | | | 0.002 | 0.035 |
| Ever treated for nausea or vomiting of pregnancy | | | | |
| 0 | 51 | 46 | 1.00 | 1.00 |
| 1–3 | 31 | 25 | 1.12 | 1.14 (0.56–2.29) |
| 4–7 | 24 | 21 | 1.03 | 0.91 (0.41–1.99) |
| ≥ 8 | 20 | 17 | 1.06 | 1.05 (0.44–2.51) |
| Trend *P* | | | 0.93 | 0.98 |
| Hormones given to suppress lactation | | | | |
| Never | 240 | 245 | 1.00 | 1.00 |
| Once | 109 | 107 | 1.04 | 0.96 (0.66–1.39) |
| Twice or more | 103 | 100 | 1.05 | 1.07 (0.69–1.67) |
| Trend *P* | | | 0.74 | 0.81 |
| Hormones given to induce or promote labour | | | | |
| Never | 277 | 278 | 1.00 | 1.00 |
| Ever | 175 | 174 | 1.01 | 1.04 (0.77–1.40) |
| *P* | | | 0.95 | 0.79 |

[a]Univariate model. [b]Multivariate model included age at first full-term pregnancy, number of full-term pregnancies, lifetime months of breastfeeding, age at menarche, first degree family history of breast cancer, lifetime months of oral contraceptive use, race, average alcohol consumption per week, and average hours per week of physical activity during reproductive years. [c]CI = confidence interval. [d]All pregnancies treated for nausea or vomiting were full term; treatments included use of drugs and/or hospitalization. [e]Model includes all variables in footnote b except lifetime months of breastfeeding.

estimates. The protective association of duration of breastfeeding with breast cancer risk was substantially greater among women who were older than among women who were younger when they first breastfed.

We analysed age at first breastfeeding separately for women who breastfed their first child from women who first breastfed a later child. Among women who breastfed their first child, there was no association of age at first breastfeeding with breast cancer risk (< 20 years of age compared with never breastfeeding (multivariate OR = 1.01, 95% CI 0.55–11.83; ≥ 30 years of age compared with never breastfeeding, multivariate OR = 1.01, 95% CI 0.62–1.65; trend $P = 0.73$ (trend test restricted to women who breastfed)). Among women who first breastfed a later child breast cancer risk decreased slightly with increasing age at first breastfeeding (< 20 years of age, multivariate OR = 1.38, 95% CI 0.20–9.69; ≥ 30 years of age, multivariate OR = 0.82, 95% CI 0.37–1.81; trend $P = 0.58$).

The association of lifetime duration of breastfeeding with breast cancer risk was weakly modified by age at menarche (*P* for interaction = 0.10) and first-degree family history of breast cancer (*P* for interaction = 0.08). Lifetime duration of breastfeeding was not associated with breast cancer risk among women with menarche below age 13 years (≥ 16 months of breastfeeding, multivariate OR = 1.17, 95% CI 0.62–2.21). However, among women with menarche at 13 years or older, breast cancer risk was decreased substantially with increasing duration of breastfeeding (≥ 16 months of breastfeeding, multivariate OR = 0.33, 95% CI 0.16–0.68). Although the number of women with a first-degree family history of breast cancer who also breastfed was too small to explore the breastfeeding–breast cancer association in a multivariate analysis, a univariate analysis revealed no duration effect (≥ 16 months of breastfeeding compared with never breastfeeding, OR = 1.25, 95% CI 0.36–4.37). Among women with no first-degree family history of breast cancer, the association of lifetime duration of breastfeeding with breast cancer risk was similar to the results for all women combined (≥ 16 months of breastfeeding, multivariate OR = 0.62, 95% CI 0.38–1.02). No evidence of effect modification by physical activity or years of oral contraceptive use on breast cancer risk was observed.

In a multivariate analysis, we found that women who had been treated for nausea or vomiting of pregnancy with drugs or hospitalization were at increased breast cancer risk compared with those who had not been so treated (Table 4). These results did not vary by age at first full-term pregnancy (not shown). We analysed the association of treatment for nausea or vomiting of pregnancy with breast cancer separately for women with a full-term pregnancy within the past 5 years and for those with a more distant pregnancy (Table 4). Among women with recent full-term pregnancy, the breast cancer risk was twofold higher for women who had been treated for nausea or vomiting of pregnancy than for women who had not been so treated. The association was greatly reduced among women who did not experience a recent full-term pregnancy. Breastfeeding did not reduce breast cancer risk of women who were treated for nausea or vomiting of pregnancy, but it substantially reduced the risk of women who were not treated for these conditions (Table 4). The findings were the same when the analysis was restricted to women who gave birth within 5 years of diagnosis (not shown).

Breast cancer risk was not associated with use of hormones to suppress lactation or exposure to hormones to induce or promote labour (Table 4).

Excluding pairs that included a patient with in situ breast cancer ($n = 40$ matched pairs) did not substantially affect the results presented (not shown).

### DISCUSSION

In this group of young, premenopausal women, longer duration of breastfeeding was associated with reduced breast cancer risk. Number of children breastfed was unassociated with risk, suggesting that the total duration of breastfeeding is most relevant to protection. These results are consistent with the results of other studies (Byers et al, 1985; Katsouyanni et al, 1986; McTiernan and Thomas, 1986; Rosero-Bixby et al, 1987; Tao et al, 1988; Yuan et al, 1988; Layde et al, 1989; Wang et al, 1992; Yoo et al, 1992; UK National CC Study Group, 1993; Yang et al, 1993; Newcomb et al,

© Cancer Research Campaign 1997

1994; Brinton et al, 1995; Romieu et al, 1996) which generally found modest protection from breast cancer with long duration of breastfeeding, especially among premenopausal women.

This protective effect of longer duration of breastfeeding was greater among women who had experienced one full-term pregnancy than among women who had experienced two or more full-term pregnancies. We thought it important from a public health perspective to determine whether the small protection observed among women with two or more full-term pregnancies was actually due to the protective effect of breastfeeding during a woman's first pregnancy. Breast cancer risk was moderately reduced among women who breastfed for 8 months or more following their second or subsequent pregnancies, suggesting that the protective effect of increased duration of breastfeeding is not restricted to the first pregnancy.

Breast stem cells differentiate during the first full-term pregnancy and first lactation rendering them less susceptible to carcinogenesis (Russo et al, 1982). However, unlike the findings of two recent studies (Newcomb et al, 1994; Brinton et al, 1995) age at first breastfeeding did not substantially modify the breast cancer–breastfeeding relationship. In fact, contrary to these studies, the protective association of duration of breastfeeding with breast cancer risk was greater among women who breastfed for the first time at older ages than among women who breastfed for the first time at younger ages. However, we have discussed the difficulty in determining whether any observed association of age at first breastfeeding is independent of an effect of age at first birth (Ross and Yu, 1994). We attempted to address this issue by evaluating risk by age at first breastfeeding for women who breastfed their first child separately compared with women who only breastfed a later child. Among women who breastfed their first child, age at first breastfeeding had no effect on breast cancer risk. The small, but statistically significant, decrease in risk with increasing age at first breastfeeding among women who breastfed only children born after their first child may indicate some independent effect on breast cancer risk from that associated with age at first birth.

The breastfeeding–breast cancer association varied somewhat by age at menarche and first-degree family history of breast cancer. The protective effect of longer durations of breastfeeding was only observed among women who had experienced an older age at menarche and who had no first-degree family history of breast cancer. These findings suggest that breastfeeding may be most protective among women who do not have these well established breast cancer risk factors.

The most likely mechanism for its effect on breast cancer risk is that breastfeeding delays the resumption of ovulation postpartum (Vorherr, 1973; Gray et al, 1990), reducing a woman's cumulative number of ovulatory cycles, thereby potentially reducing her risk of breast cancer (Henderson et al, 1985). The return of regular ovulatory cycles tends to occur more quickly when the number of breastfeedings per day is reduced through the use of supplemental feedings (Stern et al, 1986). As we did not obtain information about such supplemental feedings, we are unable to evaluate its impact on our results.

Women who experienced nausea and vomiting in pregnancy requiring treatment of their symptoms had an increased breast cancer risk. We previously reported that women with intractable nausea and vomiting of pregnancy (hyperemesis gravidarum) had, on average, 26% higher first-trimester serum oestradiol levels than women who did not vomit during pregnancy, and we hypothesized that the higher oestrogen exposure may contribute to an increased breast cancer risk (Depue et al, 1987). When we restricted the present analysis to women whose most recent pregnancy was within 5 years of diagnosis, risk was more than twofold higher among women who had been treated for nausea or vomiting of pregnancy. We did not obtain information about specific treatment regimens from the subjects, so we do not know if the observed association was limited to women receiving specific antiemetic drugs. However, the observed association probably underestimates the true risk, because we obtained information only about the most severe cases of nausea and vomiting of pregnancy; the comparison group undoubtedly included women who experienced some level of nausea or vomiting during their pregnancies but did not seek treatment. The association of breast cancer risk with treatment for nausea or vomiting of pregnancy was considerably weakened among women whose most recent pregnancy was 5 years or more before diagnosis. Breastfeeding was only associated with reduced breast cancer risk among women who were not treated for nausea and vomiting of pregnancy, regardless of the recency of the woman's last pregnancy. These findings strongly suggest that the increase in breast cancer risk associated with severe nausea and vomiting of pregnancy is transient. This in turn may be due to hormonally induced differentiation and proliferation of breast stem cells, some of which may have undergone malignant transformation (Miller, 1993). Breastfeeding appears to protect against breast cancer in the years immediately following pregnancy, perhaps by reducing the cyclic hormonal stimulation of breast cells, except among women who have experienced severe nausea and vomiting of pregnancy. The excessively elevated oestradiol levels of such women may irreversibly promote premalignant breast cells but, further research is needed to confirm this hypothesis.

The use of hormones to suppress lactation was not associated with risk of breast cancer, similar to the findings of others (Newcomb et al, 1994). Risk was also not associated with the use of hormones to induce or promote labour.

In this group of premenopausal women, many of whom were still bearing children or who had experienced pregnancies in the recent past, the protective role of breastfeeding and the adverse role of nausea and vomiting of pregnancy on breast cancer risk were greatest in the years immediately following pregnancy. These findings are relevant to the short-term increase in breast cancer risk following pregnancy. Breastfeeding can be promoted through the education and support of new mothers and might have an appreciable impact on future breast cancer incidence.

### ACKNOWLEDGEMENTS

We are grateful to all of the women who participated in this study and to the physicians who allowed us to contact their patients. We would also like to thank Rosemarie Hanisch for data collection, and Jane Sullivan-Halley for computing and statistical assistance. This study was supported by Public Health Service grants CA44546 and CA17054 from the National Cancer Institute, National Institutes of Health, Department of Health and Human Services; and by the California Public Health Foundation, subcontract 050-F-8709, which is supported by the California Department of Health Services as part of its statewide cancer-reporting program mandated by Health and Safety Code Sections 210 and 211.3. Dr Enger was supported by funds from the Breast Cancer Fund of the State of California through the Breast Cancer Research Program of the University of California, Grant Number 1FB-0341.

© Cancer Research Campaign 1997

### REFERENCES

Adami HO, Bergstrom R, Lund E and Meirik O (1990) Absence of association between reproductive variables and the risk of breast cancer in young women in Sweden and Norway. *Br J Cancer* **62**: 122–126

Albrektsen G, Heuch I and Kvale G (1995) The short-term and long-term effect of a pregnancy on breast cancer risk: a prospective study of 802 457 parous Norwegian women. *Br J Cancer* **72**: 480–484

Bernstein L, Henderson BE, Hanisch R, Sullivan-Halley J and Ross RK (1994) Physical exercise and reduced risk of breast cancer in young women. *J Natl Cancer Inst* **86**: 1403–1408

Brinton LA, Potischman NA, Swanson CA, Schoenberg JB, Coates RJ, Gammon MD, Malone KE, Stanford JL and Daling JR (1995) Breastfeeding and breast cancer risk. *Cancer Causes Control* **6**: 199–208

Bruzzi P, Negri E, La Vecchia C, Decarli A, Palli D, Parazzini F and Rosselli Del Turco M (1988) Short term increase in risk of breast cancer after first full term pregnancy. *Br Med J* **297**: 1096–1098

Byers T, Graham S, Rzepka T and Marshall J (1985) Lactation and breast cancer. Evidence for a negative association in premenopausal women. *Am J Epidemiol* **121**: 664–674

Cummings P, Stanford JL, Daling JR, Weiss NS and McKnight B (1994) Risk of breast cancer in relation to the interval since last full term pregnancy. *Br Med J* **308**: 1672–1674

Depue RH, Bernstein L, Ross RK, Judd HL and Henderson BE (1987) Hyperemesis gravidarum in relation to estradiol levels, pregnancy outcome, and other maternal factors: a seroepidemiologic study. *Am J Obstet Gynecol* **156**: 1137–1141

Gray RH, Campbell OM, Apelo R, Eslami SS, Zacur H, Ramos RM, Gehret JC and Labbok MH (1990) Risk of ovulation during lactation. *Lancet* **335**: 25–29

Henderson BE, Ross RK, Judd HL, Krailo MD and Pike MC (1985) Do regular ovulatory cycles increase breast cancer risk? *Cancer* **56**: 1206–1208

Hsieh C-C, Pavia M, Lambe M, Lan SJ, Colditz GA, Ekbom A, Adami HO, Trichopoulos D and Willett WC (1994) Dual effect of parity on breast cancer risk. *Eur J Cancer* **30A**: 969–973

Katsouyanni K, Trichopoulos D, Boyle P, Xirouchaki E, Trichopoulou A, Lisseos B, Vasilaros S and MacMahon B (1986) Diet and breast cancer: a case-control study in Greece. *Int J Cancer* **38**: 815–820

Lambe M, Hsieh C-C and Trichopoulos D, Ekbom A, Pavia M and Adami HO (1994) Transient increase in the risk of breast cancer after giving birth. *N Engl J Med* **331**: 5–9

Layde PM, Webster LA, Baughman AL, Wingo PA, Rubin GL, Ory HW and the Cancer and Steroid Hormone Study Group (1989) The independent associations of parity, age at first full term pregnancy, and duration of breastfeeding with the risk of breast cancer. *J Clin Epidemiol* **42**: 963–973

Leon DA, Carpenter LM, Broeders MJM, Gunnarskog J and Murphy MFG (1995) Breast cancer in Swedish women before age 50: evidence of a dual effect of completed pregnancy. *Cancer Causes Control* **6**: 283–291

McTiernan A and Thomas DB (1986) Evidence for a protective effect of lactation on risk of breast cancer in young women. *Am J Epidemiol* **124**: 353–358

Miller WR (1993) Hormonal factors and risk of breast cancer. *Lancet* **341**: 25–26

Newcomb PA, Storer BE, Longnecker MP, Mittendorf R, Greenberg ER, Clapp RW, Burke KP, Willett WC and MacMahon B (1994) Lactation and a reduced risk of premenopausal breast cancer. *N Engl J Med* **330**: 81–87

Romieu I, Hernandez-Avila M, Lazcano E, Lopez L and Romero-Jaime R (1996) Breast cancer and lactation history in Mexican women. *Am J Epidemiol* **143**: 543–552

Rosero-Bixby L, Oberle MW and Lee NC (1987) Reproductive history and breast cancer in a population of high fertility, Costa Rica, 1984–85. *Int J Cancer* **40**: 747–754

Ross RK and YU MC (1994) (letter) *N Engl J Med* **330**: 1683

Russo J, Tay LK and Russo I (1982) Differentiation of the mammary gland and susceptibility to carcinogenesis. *Breast Cancer Res Treat* **2**: 5–73

Stern JM, Konner M, Herman TM and Reichlin S (1986) Nursing behavior; prolactin and postpartum amenorrhea during prolonged lactation in American and !Kung mothers. *Clin Endocrinol* **25**: 247–258

Tao SC, YU MC, Ross RK and Xiu KW (1988) Risk factors for breast cancer in Chinese women in Beijing. *Int J Cancer* **42**: 495–498

United Kingdom National Case–Control Study Group (1993) Breastfeeding and risk of breast cancer in young women. *BMJ* **307**: 17–20

Vatten LJ and Kvinnsland S (1992) Pregnancy-related factors and risk of breast cancer in a prospective study of 29,981 Norwegian women. *Eur J Cancer* **28A**: 1148–1153

Vorherr H (1973) Contraception after abortion and postpartum. *Am J Obstet Gynecol* **117**: 1002–1025

Wang QS, Ross RK, Yu MC, Ning JP, Henderson BE and Kimm HT (1992) A case-control study of breast cancer in Tianjin, China. *Cancer Epidemiol Biomarkers Prev* **1**: 435–439

Williams EMI, Jones L, Vessey MP and McPherson K (1990) Short term increase in risk of breast cancer associated with full term pregnancy. *Br Med J* **300**: 578–579

Woods KL, Smith SR and Morrison JM (1980) Parity and breast cancer: evidence of a dual effect. *Br Med J* **281**: 419–421

Yang CP, Weiss NS, Band PR, Gallagher RP, White E and Daling JR (1993) History of lactation and breast cancer risk. *Am J Epidemiol* **138**: 1050–1056

Yoo KY, Tajima K, Kuroishi T, Hirose K, Yoshida M, Miura S and Murai H (1992) Independent protective effect of lactation against breast cancer: a case-control study in Japan. *Am J Epidemiol* **135**: 726–733

Yuan JM, Yu MC, Ross RK, Gao YT and Henderson BE (1988) Risk factors for breast cancer in Chinese women in Shanghai. *Cancer Res* **48**: 1949–1953

© Cancer Research Campaign 1997